# Female mice lacking *Pald1* exhibit endothelial cell apoptosis and emphysema

**DOI:** 10.1038/s41598-017-14894-9

**Published:** 2017-11-13

**Authors:** Isabel Egaña, Hiroshi Kaito, Anja Nitzsche, Lore Becker, Carolina Ballester-Lopez, Colin Niaudet, Milena Petkova, Wei Liu, Michael Vanlandewijck, Alexandra Vernaleken, Thomas Klopstock, Helmut Fuchs, Valerie Gailus-Durner, Martin Hrabe de Angelis, Helge Rask-Andersen, Henrik J. Johansson, Janne Lehtiö, Liqun He, Ali Ö. Yildirim, Mats Hellström, German Mouse Clinic Consortium, Antonio Aguilar-Pimentel, Markus Ollert, Carsten Schmidt-Weber, Oana Amarie, Jochen Graw, Johannes Beckers, Lillian Garrett, Sabine M. Hölter, Annemarie Zimprich, Wolfgang Wurst, Kristin Moreth, Raffi Bekeredjian, Frauke Neff, Julia Calzada-Wack, Ildikó Rácz, Andreas Zimmer, Birgit Rathkolb, Eckhard Wolf, Jan Rozman, Martin Klingenspor, Tobias Stoeger, Oliver Eickelberg, Irina Treise, Dirk H. Busch, Manuela Östereicher, Ralph Steinkamp, Christoph Lengger, Holger Maier, Claudia Stoeger, Stefanie Leuchtenberger

**Affiliations:** 2 German Mouse Clinic, Institute of Experimental Genetics, Helmholtz Zentrum München, German Research Center for Environmental Health (GmbH), Neuherberg, Germany; 4 Comprehensive Pneumology Center, Institute of Lung Biology and Disease, Helmholtz Zentrum München, German Research Center for Environmental Health (GmbH), The German Center for Lung Research (DZL), Munich, Germany; 6 German Center for Vertigo and Balance Disorders, Munich, Germany; 7 Deutsches Zentrum für Neurodegenerative Erkrankungen e. V. (DZNE), Munich, Germany; 9 Experimental Genetics, Center of Life and Food Sciences Weihenstephan, Technical University of Munich, Neuherberg, Germany; 10 German Center for Diabetes Research (DZD), Neuherberg, Germany; 13 Department of Infection and Immunity, Luxembourg Institute of Health, Esch-sur-Alzette, Luxembourg, and Department of Dermatology and Allergy Center, Odense Research Center for Anaphylaxis, University of Southern Denmark, Odense, Denmark; 14 Center of Allergy & Environment (ZAUM), Technische Universität München, and Helmholtz Zentrum München, Ingolstädter Landstrasse, 85764 Neuherberg, Germany; 15 Institute of Developmental Genetics, Helmholtz Zentrum München, German Research Center for Environmental Health GmbH, Ingolstädter Landstrasse 1, 85764 Neuherberg, Germany; 16 Developmental Genetics, Center of Life and Food Sciences Weihenstephan, Technische Universität München, Ingolstädter Landstrasse 1, 85764 Neuherberg, Germany; 17 Munich Cluster for Systems Neurology (SyNergy), Adolf-Butenandt-Institut, Ludwig-Maximilians-Universität München, Schillerstrasse 44, 80336 Munich, Germany; 18 Department of Cardiology, University of Heidelberg, Im Neuenheimer Feld 410, 69120 Heidelberg, Germany; 19 Institute of Pathology, Helmholtz Zentrum München, German Research Center for Environmental Health GmbH, Ingolstädter Landstrasse 1, 85764 Neuherberg, Germany; 20 Institute of Molecular Psychiatry, University of Bonn, Sigmund-Freud-Strasse 25, 53127 Bonn, Germany; 21 Ludwig-Maximilians-Universität München, Gene Center, Institute of Molecular Animal Breeding and Biotechnology, Feodor-Lynen Strasse 25, 81377 Munich, Germany; 22 Molecular Nutritional Medicine, Technische Universität München, Else Kröner-Fresenius Center for Nutritional Medicine, 85350 Freising, Germany; 23 ZIEL – Center for Nutrition and Food Sciences, Technische Universität München, 85350 Freising, Germany; 24 Institute for Medical Microbiology, Immunology and Hygiene, Technical University of Munich, Trogerstrasse 30, 81675 Munich, Germany; 1 Science for life laboratory, Department of Immunology, Genetics and Pathology, The Rudbeck laboratory, Uppsala University, Uppsala, Sweden; 3 Department of Neurology, Friedrich-Baur-Institut, Ludwig-Maximilians-Universität München, Munich, Germany; 5 Department of Surgical Sciences, Head and Neck Surgery, Section of Otolaryngology, Uppsala University Hospital, Uppsala, Sweden; 8 German Network for Mitochondrial Disorders (mitoNET), Munich, Germany; 11 Cancer Proteomics Mass Spectrometry, Science for Life Laboratory, Department of Oncology-Pathology, Karolinska Institutet, Stockholm, Sweden; 12 Present Address: INSERM U970, 56 rue Leblanc, F-75015 Paris, France

## Abstract

Paladin (*Pald1*, *mKIAA1274* or x99384) was identified in screens for vascular-specific genes and is a putative phosphatase. Paladin has also been proposed to be involved in various biological processes such as insulin signaling, innate immunity and neural crest migration. To determine the role of paladin we have now characterized the *Pald1* knock-out mouse in a broad array of behavioral, physiological and biochemical tests. Here, we show that female, but not male, *Pald1* heterozygous and homozygous knock-out mice display an emphysema-like histology with increased alveolar air spaces and impaired lung function with an obstructive phenotype. In contrast to many other tissues where *Pald1* is restricted to the vascular compartment, *Pald1* is expressed in both the epithelial and mesenchymal compartments of the postnatal lung. However, in *Pald1* knock-out females, there is a specific increase in apoptosis and proliferation of endothelial cells, but not in non-endothelial cells. This results in a transient reduction of endothelial cells in the maturing lung. Our data suggests that *Pald1* is required during lung vascular development and for normal function of the developing and adult lung in a sex-specific manner. To our knowledge, this is the first report of a sex-specific effect on endothelial cell apoptosis.

## Introduction

Paladin or Pald1 is a phosphatase-domain containing protein and we have identified it in two independent screens for novel regulators of angiogenesis and vascular function^1,2^. Pald1 expression is prominent in developing endothelial cells during early embryonic development and, in certain vascular beds, Pald1 expression shifts to mural cells as the vasculature matures, e.g. in the CNS^3^. Furthermore, Pald1 was also found in hematopoietic cells and other non-vascular cells, such as neural crest cells^3,4^. A role of *Pald1* as a regulator for neural crest cell formation and migration in the chick embryo has been shown by morpholino knock-down experiments. It was suggested that Pald1 would not require any catalytic activity for its role in neural crest migration since mutation of the putative catalytic cysteine to serine still affected neural crest migration after over expression^4^.

Pald1 has also been shown to negatively regulate expression and phosphorylation of the insulin receptor, as well as phosphorylation of its downstream target kinase Akt in cell culture, even though no phosphatase activity could be detected *in vitro* ^5^. Pald1 has therefore been proposed to be a pseudo-phosphatase, and as such been implicated to indirectly regulate cell signaling^5–7^. However, others have predicted that Pald1 can possess catalytic activity, even though this have not been shown experimentally yet^8^. Furthermore, Pald1 has been suggested to affect another type of receptor signaling, acting as a negative regulator of Toll-like receptor 9^9^.

While there is a strong expression of *Pald1* in both the prenatal and adult lung^3^, its role in lung development or function is unknown. A central role for angiogenesis has been proposed for lung diseases like chronic obstructive pulmonary disease (COPD) and emphysema^10^. COPD is a chronic progressive disease characterized by clinical symptoms such as dyspnea and coughing, due to obstruction of the airways. COPD is a major cause of death worldwide and it is estimated that COPD will become the third leading cause of death by 2020^11,12^. COPD is frequently accompanied by emphysema, i.e. the destruction of the alveolar walls and the consequential dilation of the distal airways^10^. Alterations of mRNA and protein expression of vascular endothelial growth factor A (VEGF-A) and its receptor VEGF receptor 2 (VEGFR2) are associated with patients exhibiting COPD and emphysema^13^. Experimental disruption of VEGF-A/VEGFR2 signaling has also been shown to be sufficient to induce emphysema and COPD-like changes in mice and rats through apoptosis^14–16^. There are sex-specific differences in COPD and emphysema in humans where subsets of women develop more severe emphysema than men. Female smokers with early onset COPD or severe emphysema have smoked significantly less than their male counterparts^17^. It has also been shown that female mice develop earlier and more severe emphysema than male mice in response to cigarette smoke^18^. However, the mechanisms responsible for this apparent sex-linkage remain unclear.

Here we describe the phenotype of *Pald1* ^+/−^ and *Pald1* ^−/−^ mice^19,20^ and characterize lung defects linking *Pald1* to sex-specific endothelial cell apoptosis, development of emphysema and COPD-like changes in females. To our knowledge, this is the first report of a sex-specific effect on endothelial cell apoptosis.

## Results

### *Pald1*^−/−^ mice exhibit an obstructive lung phenotype

In order to characterize the *Pald1* ^−/−^ mouse, we performed a wide array of behavioral, physiological and biochemical tests at the German Mouse Clinic (GMC). Although most of the results turned out normal for *Pald1* ^−/−^ mice (Table S1 and Figure S1), it was noteworthy that female, but not male, *Pald1* ^−/−^ mice exhibited increased lung volumes and compliance and decreased resistance, which are changes also observed in elastase-induced emphysema mouse model^21^ (Table 1).

**Table 1:** Functional lung tests in 20-week old *Pald1* ^+/+^ and *Pald1* ^−/−^ mice. Lung function was assessed by a forced maneuver system and a Fine-Pointe RC system. Differences between genotypes were evaluated by Wilcoxon test. Data are presented as median values ± interquartile range.

	*Female*			Male		
	*Pald1* ^+/+^ (n = 6)	*Pald1* ^−/−^ (n = 6)	p-value	*Pald1* ^+/+^ (n = 7)	*Pald1* ^−/−^ (n = 5)	p-value
	Median	Median		Median	Median	
	[25%; 75%]	[25%; 75%]		[25%; 75%]	[25%; 75%]	
Body weight (g)	**26**.**2**	**25**.**6**	0.623	**34**.**9**	**33**.**4**	0.093
	[24.4, 27.7]	[23.6, 27.4]		[34.1, 36.4]	[32.8, 33.0]	
Tidal volume (ml)	**0**.**21**	**0**.**22**	0.056	**0**.**19**	**0**.**20**	0.374
	[0.21, 0.21]	[0.22, 0.22]		[0.18, 0.21]	[0.20, 0.20]	
Inspiratory capacity (ml)	**0**.**695**	**0**.**885**	**0**.**026**	**0**.**871**	**1**.**340**	**0**.**026**
	[0.650, 0.755]	[0.832, 0.982]		[0.650, 0.755]	[1.320, 1.253]	
Expiratory Reverse Volume (ml)	**0**.**26**	**0**.**29**	0.323	**0**.**24**	**0**.**26**	0.425
	[0.25, 0.29]	[0.28, 0.30]		[0.21, 0.26]	[0.24, 0.28]	
Vital Capacity (ml)	**0**.**97**	**1**.**17**	**0**.**026**	**1**.**23**	**1**.**09**	0.779
	[0.90, 1.01]	[1.12, 1.29]		[0.90, 1.46]	[1.12, 1.29]	
Functional Residual Capacity (ml)	**0**.**295**	**0**.**310**	0.370	**0**.**325**	**0**.**295**	0.980
	[0.290, 0.308]	[0.292, 0.328]		[0.329, 0.369]	[0.275, 0.365]	
Residual Volume (ml)	**0**.**025**	**0**.**035**	0.649	**0**.**029**	**0**.**056**	0.649
	[0.020, 0.053]	[0.030, 0.040]		[0.025, 0.035]	[0.030, 0.040]	
Total lung capacity (ml)	**1**.**005**	**1**.**190**	**0**.**022**	**1**.**472**	**1**.**695**	0.096
	[0.962, 1.032]	[1.137, 1.302]		[1.362, 1.532]	[1.137, 1.859]	
Forced Vital Capacity (ml)	**0**.**895**	**1**.**065**	**0**.**026**	**1**.**099**	**1**.**160**	0.323
	[0.828, 0.925]	[1.015, 1.175]		[0.828, 1.125]	[1.015, 1.175]	
Forced Expiratory Volume in 100 ms (ml)	**0**.**885**	**1**.**030**	**0**.**028**	**1**.**058**	**1**.**097**	0.507
	[0.802, 0.915]	[0.982, 1.145]		[0.976, 1.129]	[0.872, 1.201]	
Peak Expiratory Flow (ml/sec)	**30**.**1**	**30**.**7**	0.141	**33**.**6**	**31**.**7**	0.092
	[30.1, 30.5]	[30.6, 30.9]		[33.1, 34.5]	[30.8, 32.7]	
Static lung compliance (ml/cm H_2_O)	**0**.**050**	**0**.**070**	**0**.**039**	**0**.**056**	**0**.**053**	0.384
	[0.050, 0.058]	[0.063, 0.070]		[0.051, 0.059]	[0.051, 0.055]	
Dynamic lung compliance (ml/cm H_2_O)	**0**.**02**	**0**.**03**	**0**.**015**	**0**.**04**	**0**.**03**	0.370
	[0.02, 0.02]	[0.03, 0.03]		[0.03, 0.05]	[0.03, 0.03]	
Resistance (cm H_2_O/ml/sec)	**1**.**40**	**1**.**27**	**0**.**013**	**0**.**98**	**1**.**11**	0.652
	[1.35, 1.45]	[1.23, 1.31]		[0.96, 1.11]	[1.01, 1.31]	

### Paladin is expressed broadly in the adult lung

We have previously shown that Pald1 protein is abundant in the mesenchymal compartment of embryonic lungs and that *Pald1* is also highly expressed in the adult lung^3^. Single cell sorting and sequencing from mouse embryonic lung strongly supports the conclusion that *Pald1* is exclusively expressed in endothelial cells during embryonic development^22,23^.

**Figure 1:**
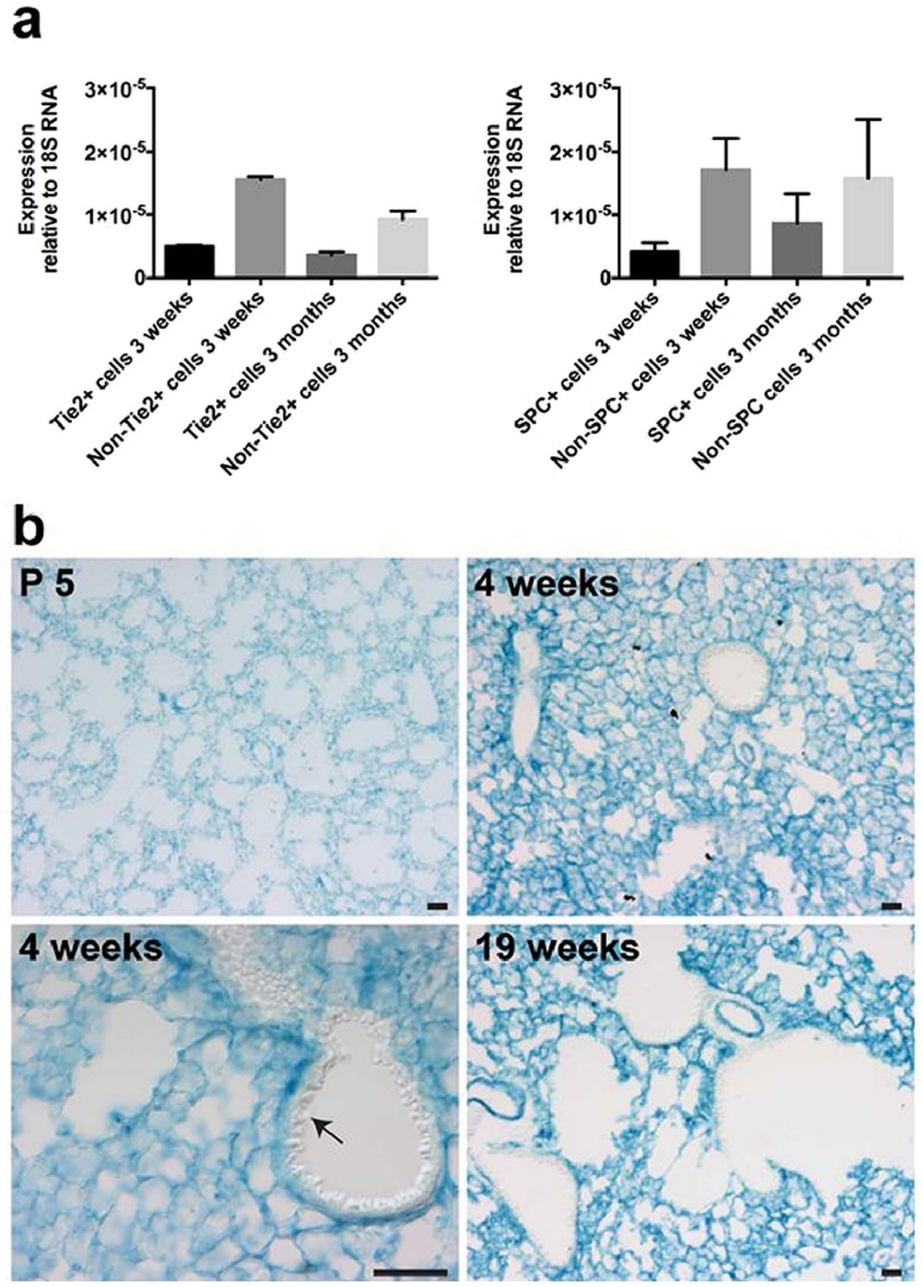
Paladin has a broad expression in the postnatal lung. (**a**) Endothelial cells and pneumocytes type II were isolated from lungs of mT/mG mice expressing endothelial-specific Tie2-Cre (left) or pneumocyte type II-specific SPC-Cre (right). Q-PCR of sorted single cells indicates *Pald1* mRNA expression in both endothelial cells and non-endothelial cells, including pneumocytes type II, both at 3 weeks and 3 months of age. Expression was normalized to 18 S RNA. (**b**) *Pald1* LacZ reporter activity (blue) is detected in *Pald1* ^LacZ/LacZ^ mice, 5 days, 4 weeks and 19 weeks after birth. LacZ is broadly expressed in the lung tissue, except for the bronchial epithelium, which shows no reporter activity (arrow). Scale bar = 200 µm.

**Figure 2:**
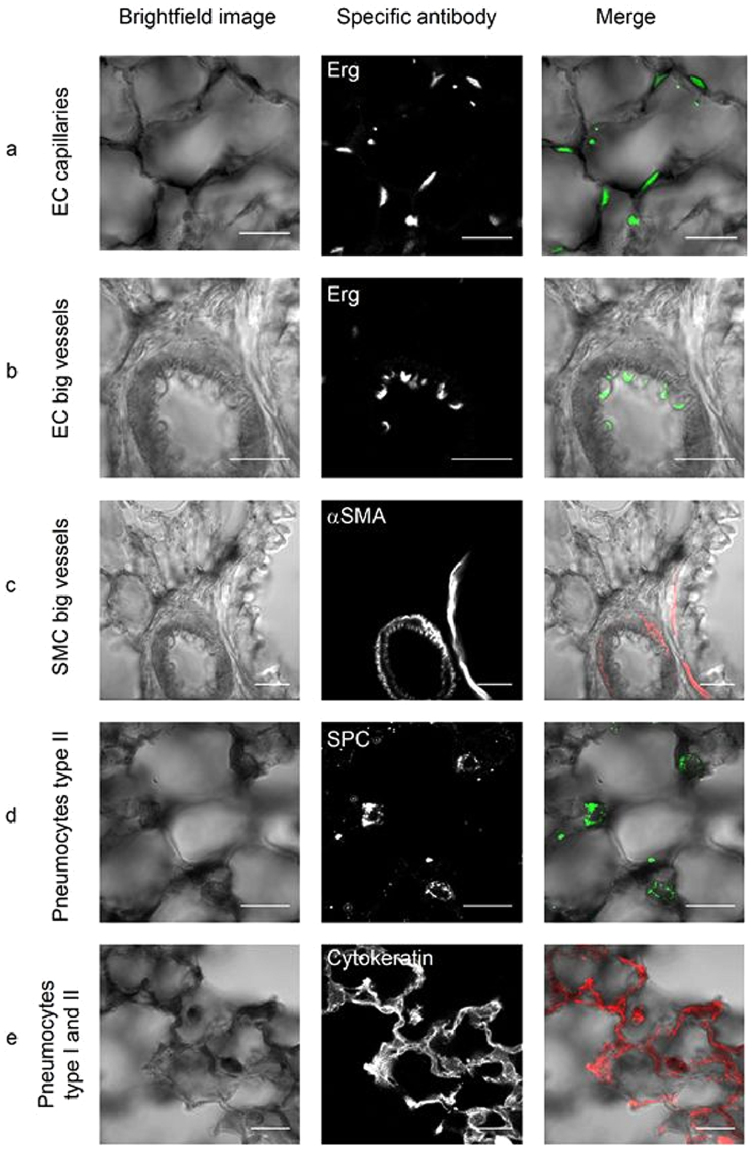
Paladin is expressed both in the epithelial and mesenchymal compartment of the postnatal lung (4 weeks). (**a**–**e**) Combined X-gal (black) and immunofluorescence staining of *Pald1* ^LacZ/LacZ^ mice show *Pald1* LacZ expression in the vasculature, i.e. endothelial cells in capillaries (**a**) but to a lesser extent in endothelial cells of larger blood vessels (**b**) as indicated by Erg staining (endothelial cell nuclei, green). In large blood vessels LacZ expression can be detected in vascular smooth muscle cells (**c**, α-smooth muscle actin, red). Paladin LacZ reporter is also active in pneumocytes type II (**d**, SPC, green) and pneumocytes type I/II (**e**, cytokeratin, red). Scale bar = 20 µm.

To begin to understand the role of *Pald1* in postnatal lung development and function, we mapped *Pald1* expression in detail. We analyzed the expression at three distinct time points during postnatal development and in the adult, postnatal day 5 (P5), 4 and 19 weeks of age as it covers main aspects of postnatal development of the major gas exchange unit of the lungs – the alveoli. The tremendous increase in gas exchange surface that occurs postnatally by formation of alveoli is accelerated from P5 until 2 weeks of age and peaks at 5–6 weeks. It is then stable until 40 weeks of age, and subsequently there is a gradual loss of alveoli. The decrease in alveolar septal thickness, that also facilitates gas exchange, continues from P5 until 4 weeks of age. Therefore, we decided to map the expression of *Pald1* and assess the phenotype of *Pald1* ^−/−^ animals at P5, 4 and 19 weeks of age, to cover the dynamic process of postnatal lung development^24^. First we isolated single endothelial cells and pneumocytes type II from lungs using mT/mG reporter mice expressing Cre-recombinase under the endothelial Tie2 promoter and under the pneumocyte type II-specific surfactant protein C (SPC) promoter, respectively^25,26^. mRNA isolation and quantitative PCR for *Pald1* showed that *Pald1* is expressed in endothelial cells, but also in type II pneumocytes (Fig. 1a). To map the expression of *Pald1* at cellular resolution, we took advantage of the LacZ reporter of *Pald1* knock-out construct^3^. Murine *Pald1* heterozygous (*Pald1* ^LacZ/+^) and homozygous mutant (*Pald1* ^LacZ/LacZ^) lungs were harvested at postnatal day (P) 5, week 4 and week 19. The LacZ reporter indicated a broad expression of *Pald1* in the murine lung in both males and females across all ages analyzed apart from bronchiolar epithelium. LacZ expression pattern was the same in *Pald1* ^LacZ/+^ and *Pald1* ^LacZ/LacZ^ lungs (Fig. 1b and Figure S2). Combining LacZ staining with antibodies specific for each of the main cell types in the alveoli revealed that *Pald1* can be detected at all stages in the endothelial cells forming the capillaries around the alveoli but is less frequently detected in the endothelium of larger vessels (Fig. 2, and Figure S3). Paladin was also detected in vascular smooth muscle cells, macrophages, pneumocytes type I and II and Platelet-derived Growth Factor Receptor α (PDGFRα)-positive mesenchymal cells (Fig. 2). However, the surfactant protein positive type II pneumocytes were not LacZ positive at P5.

Taken together, *Pald1* mRNA and protein are strongly expressed in endothelial cells as well as in other cell types in the postnatal lung, except for bronchial epithelial cells.

### Lack of *Pald1* leads to enlarged distal airspaces in female mice

**Figure 3:**
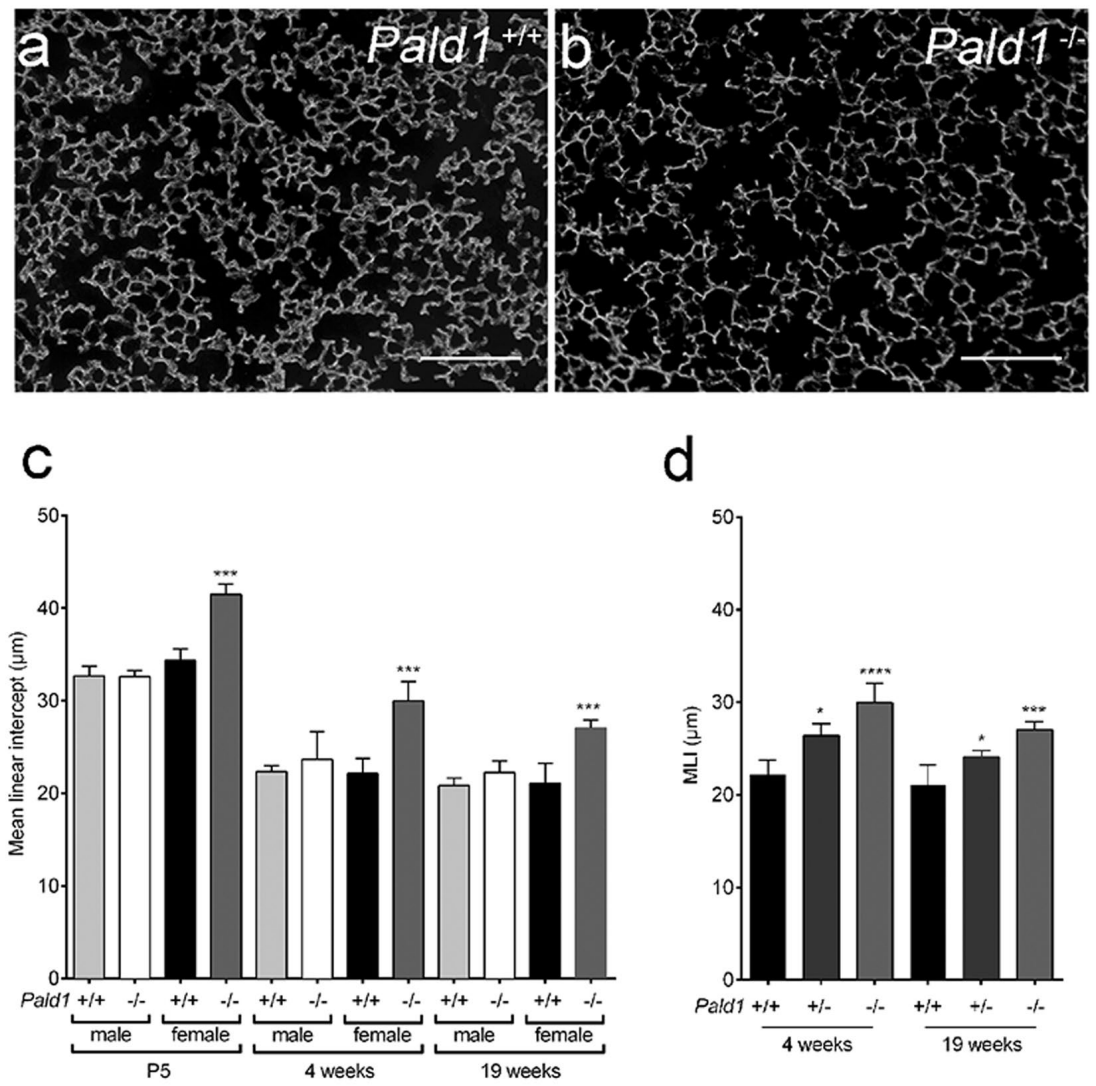
*Pald1* ^−/−^ mice show reduced complexity of the lung tissue. (**a**,**b**) Dark field images of distal airspace from 4 weeks old female *Pald1* wild type (**a**) and knock-out animals (**b**), suggest a general airspace enlargement in the *Pald1* knock-out lungs. Scale bars = 200 µm. (**c**) Quantification of interseptal alveolar distance using Mean Linear Intercept (MLI) of hematoxylin-eosin stained lung tissue at P5 (n = 3), 4 (n = 5) and 19 weeks (n = 4) shows increased air spaces in female knock-out animals at all stages, but was particularly increased at 4 weeks. ANOVA per age group: P5 p < 0.0001; 4 weeks p = 0.0002; 19 weeks p = 0.0004. *Pald1* ^−/−^ was compared to *Pald1* ^+/+^ of the same sex within each age group. (**d**) MLI of *Pald1* wild type, heterozygous and knock-out female mice at the indicated ages. ANOVA per age group: 4 weeks p = 0.0001, 19 weeks p = 0.0007. *Pald1* ^−/−^ and *Pald1* ^+/−^ were compared to *Pald1* ^+/+^ within each age group. Error bars: SD, *p ≤ 0.05, ***p ≤ 0.001, ****p ≤ 0.0001.

COPD in humans is often accompanied by emphysema, characterized by destruction of alveolar walls and distal airspace enlargement^27^. Histological analysis of *Pald1* wild type and knock-out lungs revealed enlarged distal airspaces in female mice, but not in males, assessed by both blinded lung pathologist and quantification of mean linear intercept (MLI) (Fig. 3a–c). Increase in MLI, i.e. increase in distance between airway walls, was seen already at P5, at the end of the saccular stage and before alveolarization^28^. The distal airway dilation was still present at 4 and 19 weeks of age. Interestingly, we observed that female *Pald1* ^+/−^ mice also exhibited a significant increase in distal airspace enlargement as quantified by MLI (Fig. 3d). However, no difference in alveolar septal thickness was detected (*Pald1* ^+/+^ 2,67+/− 0,14 vs. *Pald1* ^−/−^ 2,48+/−0,07 µm^3^/µm^2^, P = 0,13).

### Reduced number, increased apoptosis and proliferation of endothelial cells in female *Pald1* knock-out lungs

**Figure 4:**
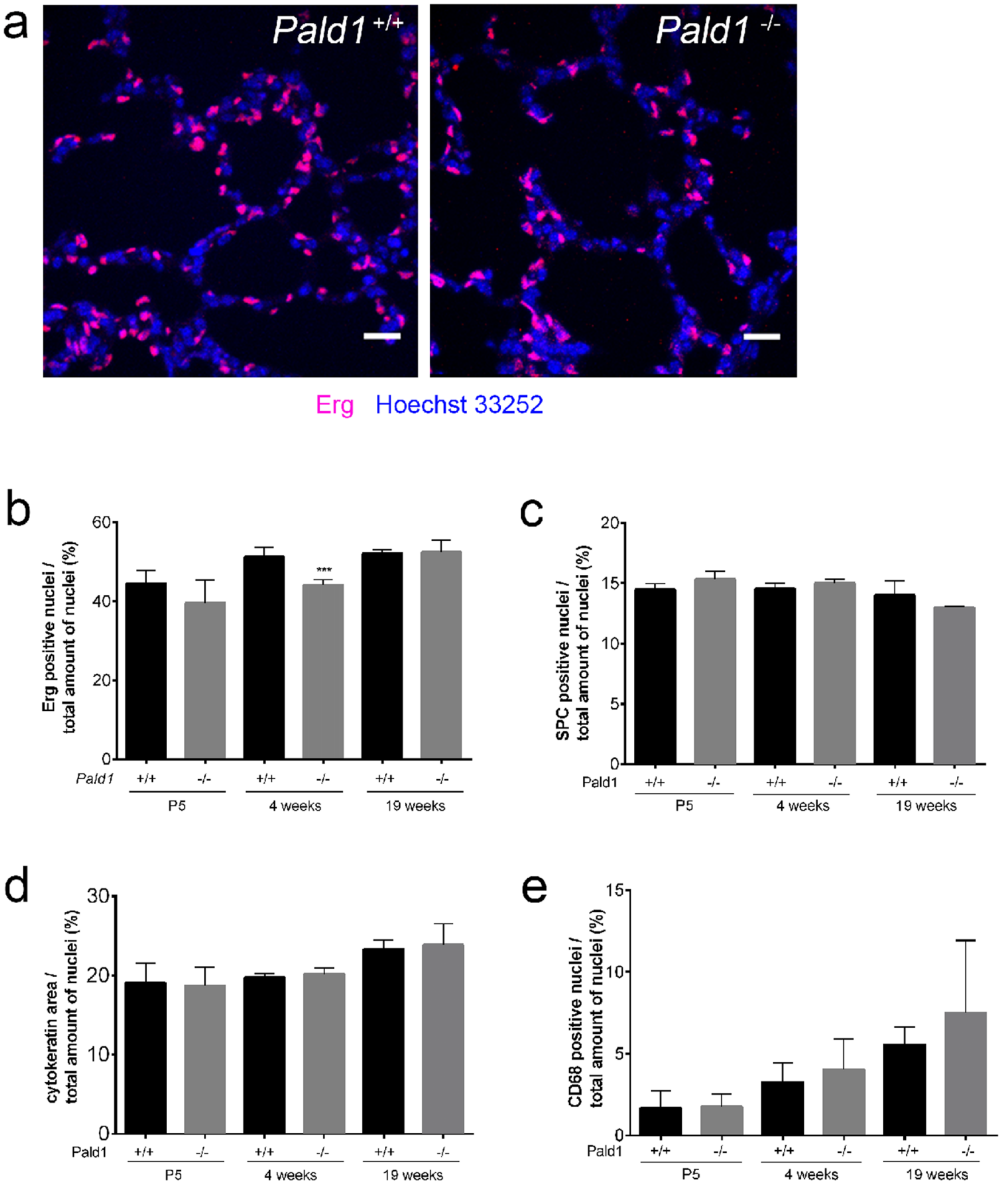
*Pald1* ^−/−^ show a decrease in the endothelial cell population at 4 weeks. (**a**–**e**) Quantification of the relative proportion of endothelial cells (**a**,**b**, Erg, n = 3–5), pneumocytes type II (**c**, SPC, n = 3), pneumocytes type I/II (**d**, cytokeratin, n = 3), and macrophages (**e**, CD68, n = 3–7). The specific cell type contribution was unaltered in *Pald1* ^−/−^ mice except for a 15% decrease of the endothelial cell population at 4 weeks in *Pald1* ^−/−^ mice. Scale bar = 20 µm, Error bars: SD, t-test between genotypes of each age group. ***p ≤ 0.001.

To further dissect the lung phenotype of female *Pald1* knock-out mice, we used cell type-specific markers to assess the relative contribution in the lung. Cell type-specific markers for pneumocytes type I or II, macrophages or PDGFRα-positive cells did not reveal any differences of cell number between wild type and *Pald1* knock-out mouse lungs over time (Fig. 4c–e and Figure S4). However, using the endothelial cell nuclei-specific marker Erg showed a reduction of endothelial cell number in the vascular compartment in females at 4 weeks of age by 14% (Fig. 4a and b).

**Figure 5:**
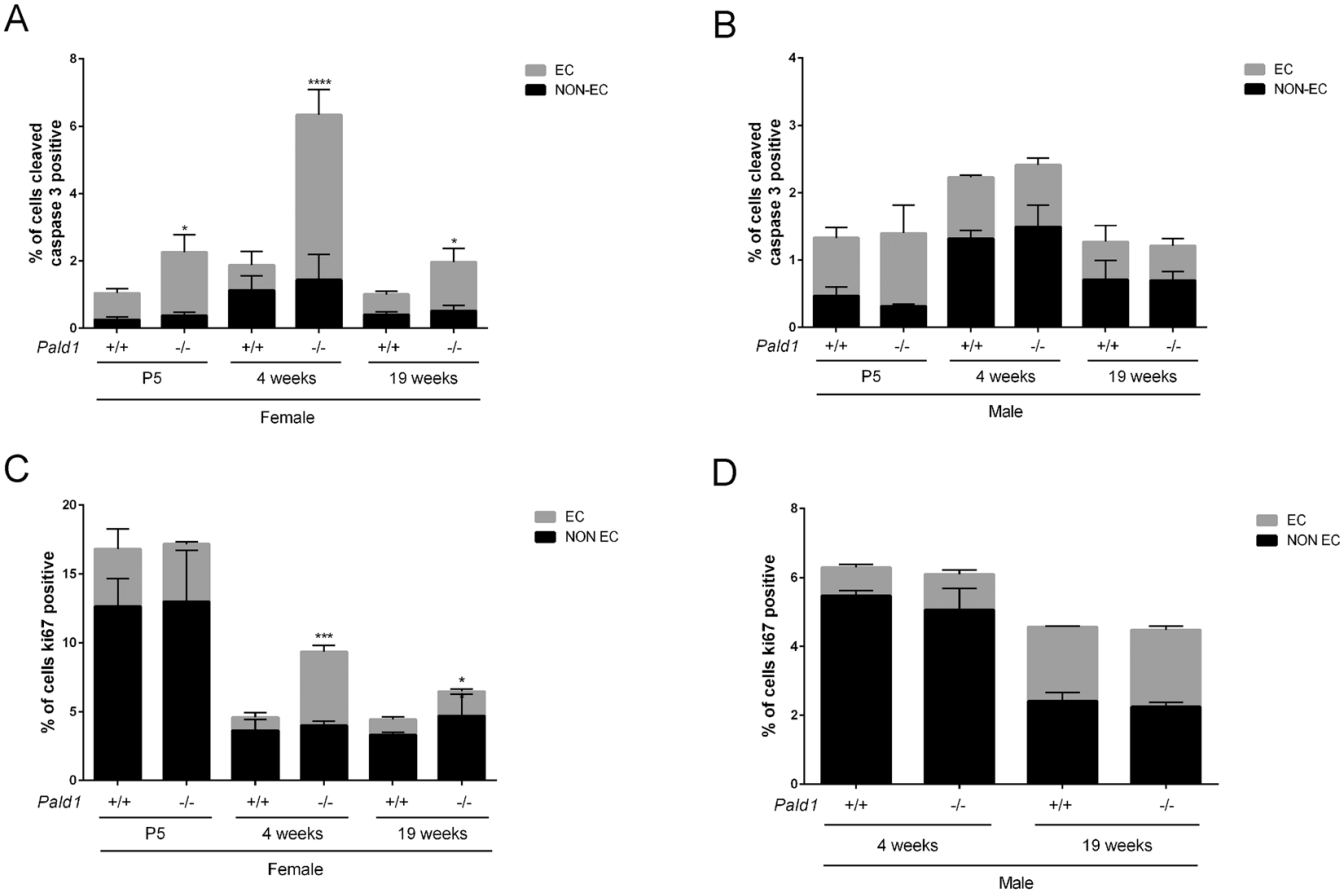
Increased apoptosis and proliferation of endothelial cells in *Pald1* knock-out female lungs. (**a**,**b**) Quantification of cleaved caspase-3 positive (CC3+) cells in 4 and 19-week old lungs revealed a significant increase in the number of cleaved caspase-3 positive endothelial cells (Erg positive) at 4 weeks of age and at 19 weeks in female (**a**, n = 3–4), but not in male *Pald1* ^−/−^ mice (**b**, n = 3–4). There was no significant increase in non-endothelial cells (Erg negative) at both 4 and 19 weeks. (**c**,**d**) Quantification of Ki67 positive cells in 4 and 19-week old lungs revealed an increased number of Ki67-positive endothelial cells (Erg positive) in female (**c**, n = 3–4), but not in male *Pald1* ^−/−^ mice (**d**, n = 3–4). There was no significant increase in non-endothelial cells (Erg negative) at both 4 and 19 weeks. Error bars: SD, t-test between genotypes within each age group. * = p ≤ 0.05, **** = p ≤ 0.0001.

Given the reduced number of endothelial cells at 4 weeks, we determined the frequency of apoptotic cells by cleaved caspase-3 (CC3) staining in both endothelial and non-endothelial cells in these lungs (Figure S5). There was a more than six-fold increase in apoptotic cells (0.75% ± 0.41% (SD) vs. 4.90% ± 0.76% CC3+ cells, p < 0.0001) in the endothelial compartment in female *Pald1* ^−/−^ compared to *Pald1* ^+/+^ mice at 4 weeks of age. However, there were no differences in non-endothelial cell apoptosis or apoptosis comparing male wild type and knock-out mice. The increase in cleaved caspase-3 was still more than two-fold at both P5 and 19 weeks of age comparing wild type and knock-out *Pald1* females (Fig. 5a and b). The enhanced endothelial apoptosis in female *Pald1* ^−/−^ was also accompanied by an increase of proliferation, as assessed by Ki-67 staining (Fig. 5c and Figure S5).

### Sex-dependent protein expression differences in lung tissue

**Figure 6:**
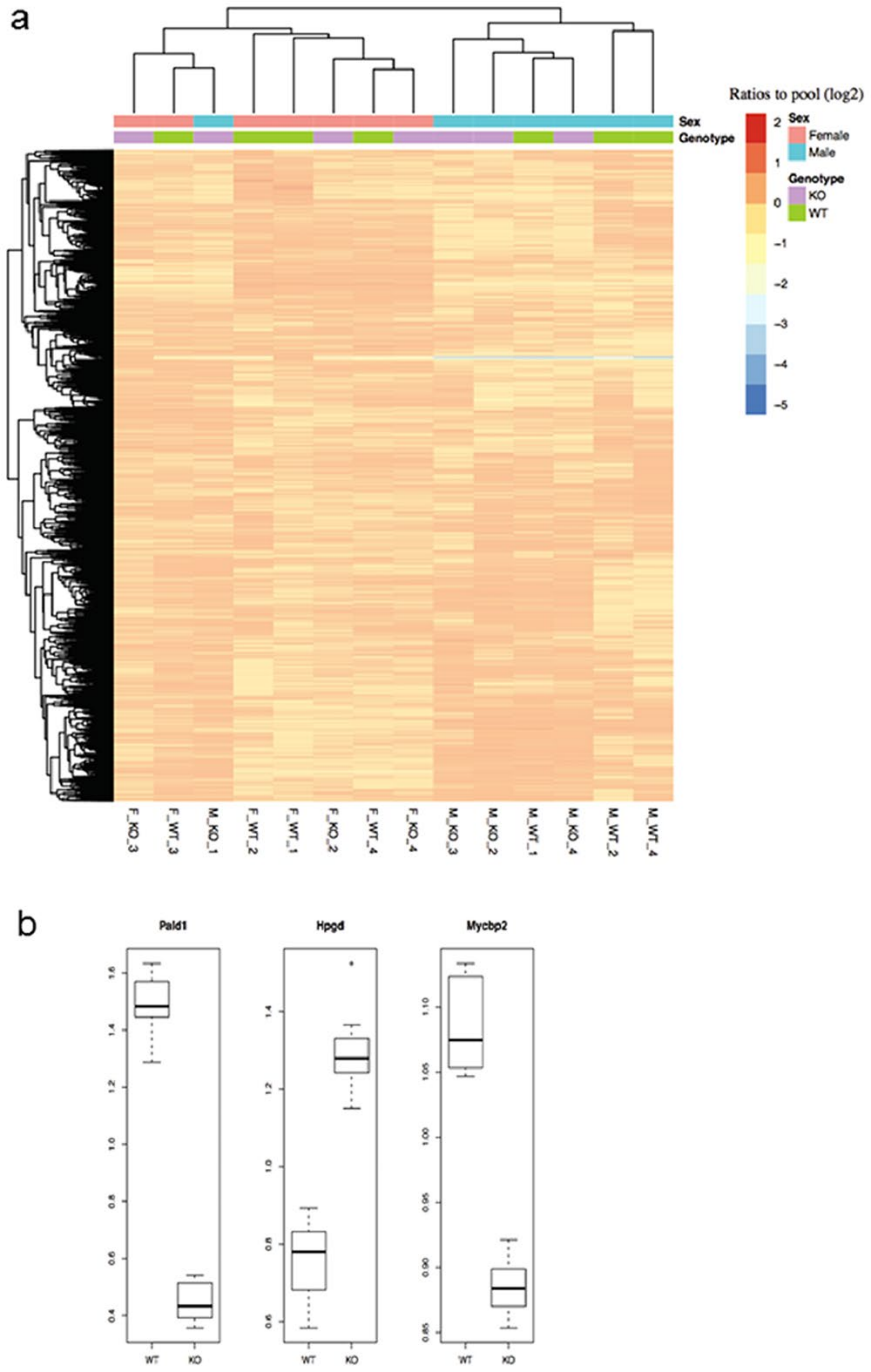
Proteomics data overview and significantly differentially expressed proteins. (**a**) Proteomics reveals sex-dependent protein expression differences in lung tissue. The heatmap shows proteome data overview of 8635 proteins with overlapping quantification in all 4 weeks of age lung samples using hierarchical clustering. Columns and rows represent lung samples and proteins, respectively. The samples are labels as sex_genotype_ID. The major factor for clustering is sex, and not genotype. (**b**) Boxplot of the three significantly differentially expressed proteins. The y-axis shows the ratios to the pool. For each gene, the ratios to the pool in the wild type group (WT) and knock-out group (KO) are plotted in the boxplot, respectively.

To begin to understand the mechanism of sex-specific phenotype in *Pald1* knock-out lungs, we employed lung proteome analysis using mass spectrometry based proteomics^29^. We identified in total 10099 proteins at 1% protein false discovery rate, and 8,635 proteins were quantified in all 16 lung samples (n = 4 per sex and genotype) from 4 weeks old mice. One female *Pald1* knock-out sample was excluded from further analyses because of contamination as described in Materials and Methods. Hierarchical clustering based on the expression of all proteins showed that the major factor for clustering is sex, and not genotype (Fig. 6a). Three proteins including Pald1 showed significantly altered expression between wild type and knock-out mice in a sex-independent manner. Hpgd (hydroxyprostaglandin dehydrogenase 15 (NAD)), showed higher expression in knock-out lungs than wild type, which was confirmed by Western blot (Fig. 6b and Figure S6). In addition, Mycbp2, MYC binding protein 2, as well as Pald1 was significantly down regulated (Figure S7). The most differentially expressed protein between wild type male and female lungs was HSD17B7, a protein essential for cholesterol biosynthesis and with the capacity to catalyze conversion of estrone to estradiol^30^. There were no statistically significant protein expression changes that were specific to female knock-out mice, after correction for multiple testing.

## Discussion

Several independent groups have identified *Pald1* as a vascular enriched gene^1,2,31^ and we have previously shown that *Pald1* is expressed in both endothelial and mural cells in the vasculature^3^.

Extensive phenotypic screening revealed morphological and functional lung defects in female *Pald1* ^−/−^ mice. This was accompanied by increased distal air spaces and elevated apoptosis and proliferation exclusively in the endothelial compartment of female mice. It is tempting to speculate, that *Pald1* has a unique role in the vasculature of the lung, as this is where we observe the strongest phenotype, even though the expression of *Pald1* is broader and we cannot exclude that the increased endothelial turnover is secondary to alteration in other cell types.

A central role for endothelial cell apoptosis in the development of emphysema has been proposed. This is based on both the correlation of increased endothelial cell apoptosis and down regulation of VEGF in human patients exhibiting emphysema^13^, and experimental evidence in animal models. Genetic ablation of VEGF-A in the lung using adenoviral Cre led to endothelial and non-endothelial cell apoptosis, without a compensatory increase in proliferation^16^. Inhibition of VEGFRs using either low molecular weight kinase inhibitors or specific VEGFR2 (but not VEGFR1) blocking antibodies was also sufficient to trigger alveolar apoptosis and development of emphysema in mice and rats^14,15^. In addition, direct induction of lung endothelial cell apoptosis using an endothelial-homing peptide triggers development of emphysema^32^, suggesting that alveolar structures cannot be maintained without endothelial cells, as well as that endothelial cell apoptosis in the lung culminates in emphysema. Comparison of MLI measurements in the above-mentioned VEGF-A/VEGFR2 targeted mice and rats shows that the emphysema development in *Pald1* ^−/−^ female mice is comparable to what is seen in those models. However, alterations of VEGF and VEGFR levels were not the reason to emphysema in the *Pald1* knock-out mice as no differences in VEGFA or VEGFR2 protein levels were detected in the proteomic analysis, or by western blot.

The increase in distal airspace in female *Pald1* knock-out mice is stable from early postnatal stage to 19 weeks of age. The mechanism of airspace enlargement must be distinct from emphysematous development in adult humans as it occurs already before alveolar septation and is non-progressive. Despite this, further studies into this phenotype, might provide additional information on what type of cellular changes and biochemical pathways that can lead to the end stage phenotype that we refer to as emphysema. Further studies will also be necessary to pinpoint the mechanism whereby *Pald1* regulates endothelial cell survival and proliferation in a sex-specific fashion. The proteomics data showed that sex, but not genotype, was the most significant factor for differential protein expression. This is consistent with the accumulating evidence regarding sex differences and lung biology^33^. Somewhat surprisingly, no specific protein expression differences were detected between female wild type and knock-out lungs. However, Hpgd, the major enzyme for degradation of prostaglandins, showed higher expression in all knock-out lungs compared to wild type, irrespective of sex. Given that prostaglandin signaling is abundant and important for lung and vascular function as well as angiogenesis^34^, it could be speculated that prostaglandin signaling is associated with the emphysema phenotype, but that the *Pald1* ^−/−^ males are somehow protected. The lack of significant protein changes between female wild type and knock-out lungs, despite of the morphological differences and endothelial apoptosis observed at the time point analyzed, could be due to several reasons including sensitivity of the proteomics screen, use of complex tissue containing several cell types and/or due to time point of analysis. Even though we observe the greatest morphological changes at 4 weeks of age, the potential protein expression differences causing those changes might have occurred earlier.

Paladin was previously identified as a negative regulator of insulin signaling by *in vitro* screening for FOXO1A-driven reporter gene expression using a human cDNA library^5^. We detected a minor reduction in the ability to clear glucose from the blood stream in male mice as assessed by an intraperitoneal glucose tolerance test (Table S1). However, this is in contrast to the reported negative effect of *Pald1* on insulin receptor signaling in cells. Further studies will be needed to determine the significance of this finding.

It was reported that *Pald1* modulated the expression of key regulatory genes in neural crest development, and plasmid mediated over expression and morpholino-based knock-down of *Pald1* delayed neural crest migration in the chick embryo^4^. Even though we have noted a prominent expression of *Pald1* in migrating neural crest cells during embryonic development^3^, we have not noted any differences in neural crest derived tissues such as cardiac outflow tract, melanocytes, cranial bones or myelination in adult *Pald1* ^−/−^ mice. However, a transient role of *Pald1* in neural crest migration during development has not been assessed.

Taken together, our comprehensive description of the *Pald1* ^−/−^ mouse revealed that the putative phosphatase *Pald1* plays a role in the development and function of the lung, and specifically in female pulmonary endothelial cell survival and proliferation. Further studies are necessary to address how the lack of *Pald1* leads to endothelial cell apoptosis and proliferation, and how that is related to the emphysema phenotype in a sex-specific manner.

## Materials and Methods

### Paladin nomenclature and mouse model

Paladin is encoded by *Pald1* (phosphatase domain containing, paladin 1, also known as x99384 or *mKIAA1274*) in mice. C57BL/6 mice with constitutive deletion for *Pald1* (Exon 1–18 replaced by a LacZ reporter cassette) have been generated^3^ and backcrossed for 10 generations. ROSA mT/mG × Tie2-Cre mice^35^ were generated as previously described^25^. ROSA mT/mG mice (Jackson Stock 007576) were crossed to Sftpc-Cre^26^ to generate ROSA mT/mG × Sftpc-Cre. All animal experiments were performed in accordance with the relevant laws and institutional guidelines and were approved by the Uppsala University board of animal experimentation. At the GMC mice were maintained in IVC cages with water and standard mouse chow according to the GMC housing conditions and German laws. All tests performed at the GMC were approved by the responsible authority of the district government of Upper Bavaria, Germany.

### FACS and quantitative PCR

Isolation of single cells from ROSA mT/mG × Tie2-Cre and ROSA mT/mG × Sftpc-Cre lungs by FACS, mRNA extraction and quantitative PCR was done as previously described^25^.

### Western blot analysis

Snap frozen lungs from 2, 3 and 22-week old mice were lysed in 20 mM HEPES, 150 mM NaCl, 1% NP40, 1x protease inhibitor cocktail (Roche), homogenized with Tissue Tearor (BioSpec Products) and sonicated six times for 5 sec at 200 W (Bioruptor, diagenode). Tissue lysates were incubated for one hour at 4 °C with rotation, and centrifuged at 21’100 g for 20 min at 4 °C. Protein concentration was measured with the BCA protein detection kit (Thermo Fisher Scientific). Lung lysates were denatured in sample buffer (Life Technologies) and proteins were separated on a 4–12% BisTris polyacrylamide gel (Novex by Life Technologies). Proteins were transferred to an Immobilon-P PVDF membrane (Millipore) using the XCell II™ Blot Module (Novex by LifeTechnologies). The membrane was blocked with 5% skimmed milk in TBS 0.1% Tween and incubated with rabbit anti-Pald1 (1:1000; Atlas Antibodies, HPA017343) or goat anti-actin (1:1000, Santa Cruz, sc-1615) antibodies overnight at 4 °C. Membranes were washed in TBS 0.1% Tween and incubated with horseradish peroxidase (HRP) conjugated secondary anti-rabbit (1:10’000, GE Healthcare) and anti-goat antibodies (1:10’000, Invitrogen), respectively. Membranes were washed in TBS 0.1% Tween and developed using ECL prime (GE Healthcare). Luminescence signal was detected by the ChemiDoc MP system (BioRad).

### X-gal staining and immunohistochemistry of lung sections

Lungs were inflated and fixed intratracheally with 4% PFA. The trachea was tied under pressure, and the lung was fixed for 2 h in 4% PFA at 4 °C. For histochemical analysis, paraffin sections of fixed lungs were dehydrated gradually in a series of 70% ethanol to xylene and soaked in paraffin (for 6 h in total) prior to embedding. Paraffin sections (6 µm) were deparaffinized using xylene, rehydrated in graded alcohol series (99.6% to 70% ethanol) and rinsed in distilled water. For H&E staining, sections were immersed sequentially in hematoxylin and eosin solutions (Histolab). Stained sections were dehydrated (70% ethanol to 99.6% ethanol, xylene) and mounted in PERTEX mounting media (Histolab). Interalveolar septal wall distance was measured by the mean linear intercept (MLI). Images from H&E stained lung sections were acquired on a Nikon light microscope (Nikon Eclipse 80i, Nikon digital camera DXM 1200) at a 400x magnification. The MLI was obtained by dividing the length of a line drawn across the lung section by the total number of intercepts encountered in 10 lines per picture. Twenty-five images per section, and two sections per lung, were analyzed. One section was from the inferior lobe and another one from the superior lobe.

For frozen lung sections, lungs were collected and fixed as described. After overnight incubation in 30% sucrose in PBS, lung sections of 5–10 µm were cut, blocked (3% BSA, 0.1% Triton x-100, 5% Normal Donkey serum [Jackson Immunoresearch], 5% Normal Mouse serum [Invitrogen] in PBS) and stained with rabbit anti-ERG (1:100, Abcam, ab92513), mouse anti-ERG (1:100, Abcam, ab140520), mouse anti-αSMA (1:100, Sigma, C6198), rabbit anti-prosurfactant Protein C (1:100, Abcam, ab40879), mouse anti-cytokeratin (1:100, Sigma, P2871), rat anti-CD68 (1:100, AbD serotec, MCA1957), rabbit anti-cleaved caspase 3 Ab-5 (1:3000, Neomarkers, RB-1611-P1), and mouse anti-ki67 (1:100, Dako, M7240) in combination with appropriate fluorophore-coupled secondary antibodies. Images were obtained with the Zeiss LSM700 confocal microscope, 63x objective and analyzed by ImageJ. For x-gal staining, frozen lung sections were post-fixed in 0.2% PFA for 10 min on ice, rinsed in PBS with 2 mM MgCl_2_ and permeabilized in detergent rinse (2 mM MgCl_2_, 0.01% sodium deoxycholate, 0.02% Nonidet P-40, PBS) for 10 min on ice, prior to overnight staining at 37 °C with 1 mg/ml x-gal (Promega) diluted in staining solution (detergent rinse containing 5 mM potassium ferricyanide, 5 mM potassium ferrocyanide). Sections were washed twice for 10 min in detergent rinse, followed by PBS.

Statistical analysis of data sets of two groups was done by Student’s t-test and of three or more groups by one-way ANOVA using GraphPad Prism6.

### Lung function analysis

Lung function analysis were performed as previously reported^36^. Briefly, lung function was assessed by a forced maneuver system and a Fine-Pointe RC system (Buxco Research Systems; Wilmington, NC, USA).

Statistical analyses were performed using R-scripts (version 3.0.2, Foundation of Statistical Computing, Vienna, Austria) implemented in the database (MausDB). Differences between genotypes were evaluated by Wilcoxon test. Statistical significance was assumed at p < 0.05. Data are presented as median values ± interquartile range.

### Proteome analysis

Mouse lung samples were lysed by SDS and prepared for mass spectrometry analysis using a modified version of the FASP protocol^29^. Peptides were labelled with TMT10plex reagent according to the manufacturer’s protocol (Thermo Scientific) and separated by immobilized pH gradient - isoelectric focusing (IPG-IEF) on 3–10 strips as described previously^29^. Extracted peptide fractions from the IPG-IEF were separated using an online 3000 RSLCnano system coupled to a Thermo Scientific Q Exactive. MSGF + Percolator in the Galaxy platform was used to match MS spectra to the Ensembl 82 mouse protein database^37^.

One of the samples in *Pald1* knock-out females was excluded from further analyses because it is highly likely that squamous epithelium could have contaminated the sample; protein expression pattern was completely different from others and some proteins specific to squamous epithelial cells had been detected. The remaining quantified proteomics data were processed in R software. To identify differentially expressed genes between different groups (knock-out group versus controls, male versus female), student’s t test was used and multiple test correction was implemented using the false discovery rate method^38^. Heatmap analysis was performed using the pheatmap packages in R software. The genes were clustered using Pearson correlation distance and the samples were clustered using Euclidean distance. The average linkage cluster method was used to build the cluster dendrogram.

### Data Availability

Mass spectrometry proteomics data is deposited to jPOSTrepo^39^ with the dataset identifier JPST000225 & PXD005625.

All other data generated or analyzed during this study are included in this published article (and its Supplementary Information files).

## Electronic supplementary material

Supplementary Information
