# Supplementary material for: Female mice lacking Pald1 exhibit endothelial cell apoptosis and emphysema
Source: Sci Rep. 2017 Nov 13;7:15453. doi: 10.1038/s41598-017-14894-9 (PMC5684320; doi:10.1038/s41598-017-14894-9)

SUPPLEMENTARY INFORMATION

Female mice lacking *Pald1* exhibit endothelial cell apoptosis and emphysema

Isabel Egaña, Hiroshi Kaito, Anja Nitzsche, Lore Becker, Carolina Ballester-Lopez, Colin Niaudet, Milena Petkova, Wei Liu, Michael Vanlandewijck, Alexandra Vernaleken, Thomas Klopstock, Helmut Fuchs, Valerie Gailus-Durner, German Mouse Clinic Consortium, Martin Hrabe de Angelis, Helge Rask-Andersen, Henrik Johansson, Janne Lehtiö, Liqun He, Ali Ö. Yildirim, and Mats Hellström

SUPPLEMENTARY INFORMATION

SUPPLEMENTARY MATERIALS & METHODS

Auditory Brainstem response

Auditory Brainstem Response (ABR) measurements were performed using a RP2.1 workstation (Tucker-Davis Technologies, TDT, USA) as previously described[1]. In short, animals were anaesthetized and electrodes were placed on the vertex (reference) and ventrolateral to the ears (active and ground) of the animals. ABR potentials, as responses to broadband clicks or pure-tone frequencies, were recorded. Frequencies ranged from 6 to 30 kHz with a 6 kHz stepping, and sound pressure levels ranged from 0 to 85 dB with 5 dB steps. Hearing thresholds were determined for each frequency as the lowest SPL producing a measurable ABR pattern response.

Number of mice analyzed per genotype: at 12 weeks of age 7-8 male and 7 female mice, at 15 weeks of age 4-5 male and 5 female mice and at 18 weeks of age 10 male and 10 female mice. For statistical analysis, a Wilcoxon Rank Sum test is used to analyze the thresholds of the different auditory stimuli.

Genomic *cdh23* analysis

For genomic *cdh23* analysis a 547 bp area spanning exon7-intron7 boundary of *cdh23* was amplified (primers: TGTGTGTCTCCCAAGGATCA; AAAGCCTGCAGCATTAGGAA) using DNA isolated from $Pald1^{+/+}$ and $Pald1^{-/-}$ tail biopsies and sequenced by Sanger Sequencing at the Uppsala Genome Center (sequencing primers: CCTCTGTCTACATTGGCCAAC; ATGACTCAGCAACACGGATG.

Analysis of PDGFRα^{+} cells

Pald1$^{+/-}$ mice bred with PDGFRα^{EGFP} mice[2] to generate Pald1$^{+/}$+ : PDGFRα$^{EGFP/+}$ mice or Pald1$^{-/-}$:PDGFRα$^{EGFP/+}$ offspring. The number of EGFP+ cells was quantified in relation to the total number of Hoechst positive cells in mages that were obtained with the Zeiss LSM700 confocal microscope, 63x objective and analyzed by ImageJ.

Western blot analysis

Snap frozen lungs from 4-week old mice were lysed in 4% (w/v) SDS, 25 mM HEPES pH 7.6 and 1mM DTT in ultrapure water. Tissues were disrupted with Tissue Tearor Homogenizer (Biospec Products) and heated at 95 °C for 5 min on a pre-warmed block. They were centrifuged for 30 minutes at maximum speed at 4 °C after sonication with Bioruptor (Diagenode, Belgium) so that supernatant could be used for western blot analysis. Lung lysates were denatured in sample buffer (Life Technologies) and proteins were separated on a 4-12 % BisTris polyacrylamide gel (Novex by Life Technologies). Proteins were transferred to an Immobilon-P PVDF membrane (Millipore) using the XCell II™ Blot Module (Novex by LifeTechnologies). The membrane was blocked with 5 % skimmed milk in TBS 0.1 % Tween and incubated with rabbit anti-Hpgd (1:1000; abcam, EPR14332-19) or mouse anti-αTubulin (1:500, Sigma, T9026) antibodies overnight at 4°C. Membranes were washed in TBS 0.1 % Tween and incubated with horseradish peroxidase (HRP) conjugated secondary anti-rabbit (1:5,000, GE Healthcare) or anti-mouse antibodies (1:5,000, Invitrogen), respectively. Membranes were washed

in TBS 0.1 % Tween and developed using ECL prime (GE Healthcare). Luminescence signal was detected by the ChemiDoc MP system (BioRad).

SUPPLEMENTARY TABLES AND FIGURES

Table S1: Overview of tests performed by the German Mouse Clinic and summary of results.

Figure S1: Altered auditory responses in *Pald1* mutant mice is likely due to variations in *Cdh23*. (a-b) Auditory Brainstem response (ABR) indicates increased hearing sensitivity in *Pald1* mutant and pure background 129SvEv mice compared to *Pald1*$^{+/+}$ and pure background C57Bl/6 mice. Mean ABR thresholds SPL (Sound pressure level) for *Pald1*$^{+/+}$ and *Pald1*$^{-/-}$ mice at 12 (n = 14-15 mice per genotype), 15 (n = 9-10 mice per genotype; significantly decreased SPL for 12-30kHz) and 18 weeks (n = 20 mice per genotype; significantly decreased SPL for 24-30kHz) of age (a) or for 12-week old *Pald1*$^{+/+}$, *Pald1*$^{+/-}$ and *Pald1*$^{-/-}$ mice as well as pure background control C57Bl/6 and 129SvEv mice (b) is given for the indicated frequencies from 6 kHz to 30 kHz. Note overlapping ABR profiles between *Pald1*$^{+/+}$ and C57Bl/6, and between *Pald1*$^{+/-}$, *Pald1*$^{-/-}$ and 129SvEv mice. Error bars: IQR. ** $p \leq 0.01$, *** $p \leq 0.001$

(c) Genomic analysis of the *Cdh23* exon 7-intron boundary demonstrated the presence of *Cdh23*^{753A} variant in *Pald1*$^{+/+}$ and pure background C57BL/6JBomTac mice. In contrast, a heterozygous or homozygous *Cdh23*^{753G} allele was present in *Pald1*$^{-/-}$ mice, which were generated on the 129SvEv background, and in the pure background 129SvEv control mice. Sequencing was performed on both DNA strands, only forward strand is shown.

Expression of *Cdh23*^{753A} leads to in-frame skipping of exon 7 and consequently results in reduced stability of cadherin 23 and hearing loss[3]. *Pald1* is within 0.6 Mb of *cdh23* on chromosome 10 and *Pald1*$^{-/-}$ mice still express the stable *Cdh23*^{753G} derived from 129SvEv mouse strain. Therefore, it is likely that the differences in hearing sensitivity observed in *Pald1* mutant mice are due to different *Cdh23* variants and not related to the specific loss of paladin expression.

Figure S2: Paladin expression in male lungs. LacZ reporter activity (blue) is detected in male *Pald1*$^{LacZ/LacZ}$ mice 4 weeks and 19 weeks after birth. LacZ is broadly expressed in the lung tissue, except for the bronchial epithelium, which shows no reporter activity (arrow). Scale bar = 50 µm

Figure S3: Paladin is expressed both in the epithelial and mesenchymal compartment of the postnatal lung (P5 and 19 weeks of age). Combined X-gal and immunofluorescence staining of *Pald1*$^{LacZ/LacZ}$ mice at P5 and 19 weeks (a - e) show *Pald1* LacZ expression in the vasculature, i.e. endothelial cells in capillaries (a) but to a lesser extent in endothelial cells of larger blood vessels (b) as indicated by Erg staining (endothelial cell nuclei, green). In large blood vessels LacZ expression can be detected in vascular smooth muscle cells (c), α-smooth muscle actin, red). Paladin LacZ reporter is not active in pneumocytes type II at P5 but is active at 19 weeks (d, SPC, green), but is present in type I/II cells (e, cytokeratin, red). Scale bar = 20 µm.

Figure S4: PDGFRα positive cells in the lung (4 -10 weeks of age). Quantification of the relative proportion of PDGFRα-EGFP positive cells with (n=2 at 4 weeks and n=1 at 10 weeks) shows no differences between wild type and *Pald1* knock-out lungs as compared to total number of cells.

Figure S5: Examples of combined immunofluorescence staining for Erg and cleaved caspase-3 or Ki-67 in wild type female mice lung at P5. A small proportion of endothelial cells (Erg, red) are also positive for cleaved caspase-3 (green) or cell cycle marker Ki-67 (green). Scale bar = 20µm.

Figure S6: Hpgd protein levels in the lung. Western blot for Hpgd in both wild type and knock-out lungs was performed at 4 weeks after birth. a-Tubulin was used as a loading control. All samples were derived at the same time and divided into two parts for different secondary detection reagents.

Figure S7: Volcano plot of the statistics result of Pald1 proteomics data. The X-axis shows the log2 scaled fold change between the Pald1 knockout and control, and Y-axis shows the –log10 scaled p values of the two group comparison. Three proteins showed statistics significant (false discovery rate < 0.05): Pald1, Mycbp2 and Hpgd.

1. Ingham, N.J., Pearson, S. & Steel, K.P. Using the Auditory Brainstem Response (ABR) to Determine Sensitivity of Hearing in Mutant Mice. in *Current Protocols in Mouse Biology* (John Wiley & Sons, Inc., 2011).
2. Hamilton, T.G., Klinghoffer, R.A., Corrin, P.D. & Soriano, P. Evolutionary divergence of platelet-derived growth factor alpha receptor signaling mechanisms. *Mol Cell Biol* **23**, 4013-4025 (2003).
3. Noben-Trauth, K., Zheng, Q.Y. & Johnson, K.R. Association of cadherin 23 with polygenic inheritance and genetic modification of sensorineural hearing loss. *Nat Genet* **35**, 21-23 (2003).

Table S1

Overview of tests performed by the German Mouse Clinic and summary of results:

Screen	Tests	Phenotype summary *Pald1*$^{-/-}$ mice
**Dysmorphology, Bone and Cartilage**	Morphological observation, Clickbox test, DXA, X-Ray	None
**Behavior**	Pre-pulse Inhibition / Acoustic Startle Reflex	Increased Acoustic Startle Reflex in male mutant mice
	Open Field	Small increase in center entry latency in mutant mice
**Neurology**	Auditory Brainstem Response	Decreased sound pressure levels, related to Cdh23^{753G} genotype from 129SvEv mouse strain due to proximity to Pald1 locus (0,6 Mb) and not Pald1 genotype
	Grip Strength, Rotarod, Modified SHIRPA, Lactate	None
**Eye Screen**	Optical Coherence Tomography	Irregular/waved like pattern of the fundic blood vessels
	Eye size, Scheimpflug, Virtual Drum, Eye Morphology	None
**Nociception**	Hotplate	None
**Metabolic Screen**	Minispec and Indirect Calorimetry (TSE)	None
**Clinical Chemistry and Hematology**	Clinical Chemistry (ad lib. fed mice), Hematology, IpGTT	IpGTT: slightly increased blood glucose concentration 30-120 min after intraperitoneal glucose injection; small changes of unclear relevance in plasma composition in male mutant mice
**Immunology Screen**	Flow Cytometry	None
**Allergy Screen**	IgE levels	None
	Transepidermal water loss	None
**Steroid Screen**	Steroid levels	None
**Cardiovascular Screen**	Awake Echocardiography, Awake Electrocardiography	Shorter QT, QTc and ST interval duration mainly in male mutant mice
**Lung Function Screen**	Lung Function	Emphysema-like phenotype in female mutant mice
**Pathology Screen**	Macroscopy, Microscopy	Differences in heart weight of females of unclear relevance

Abbreviations: DXA - Dual-energy X-ray Absorptiometry; SHIRPA - SmithKline Beecham, Harwell, Imperial College, Royal London Hospital, phenotype assessment; IpGTT - Intraperitoneal Glucose Tolerance Test;

Egana et al. Figure S1

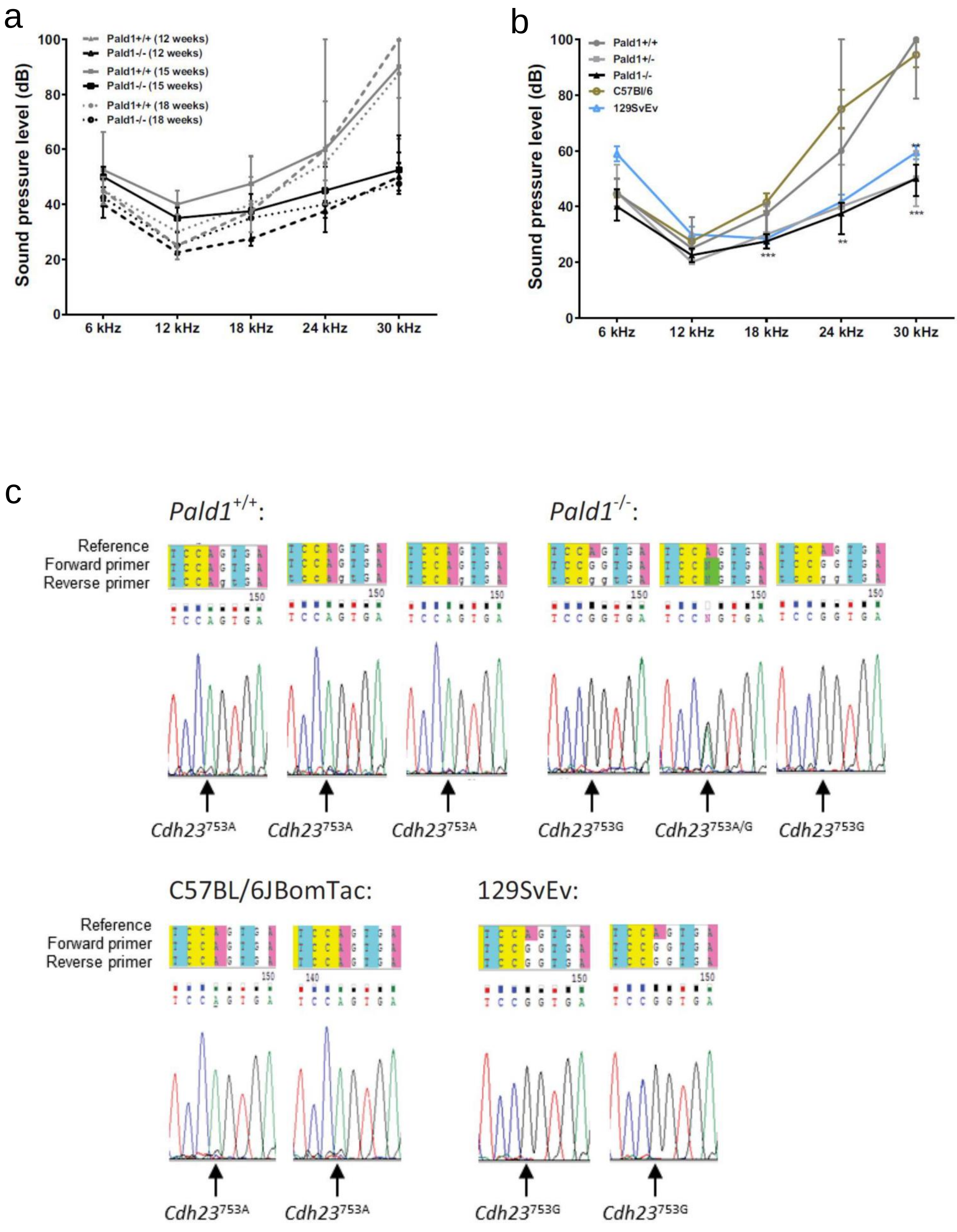

Egana et al. Figure S2

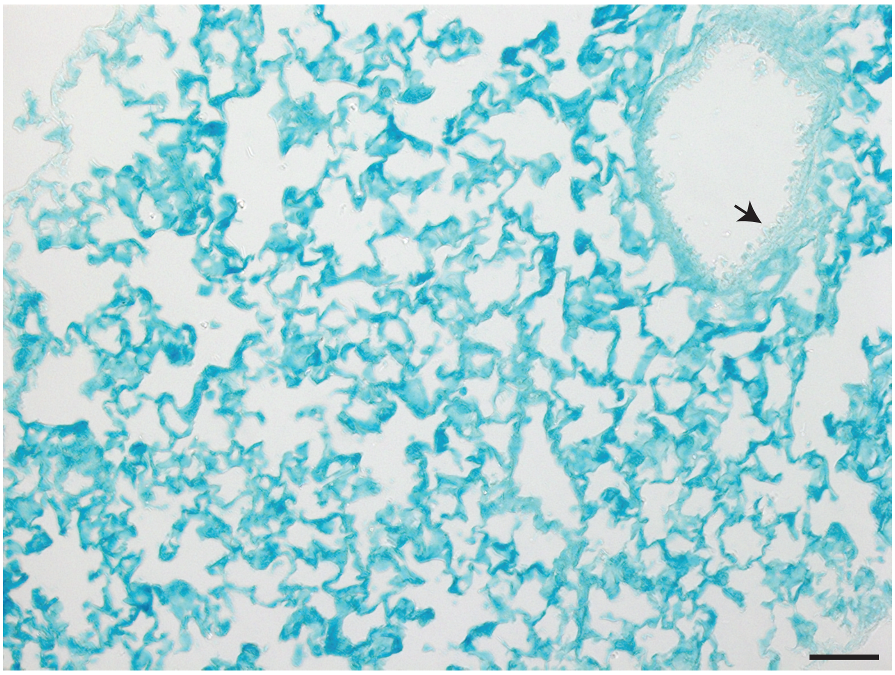

Egana et al. Figure S3

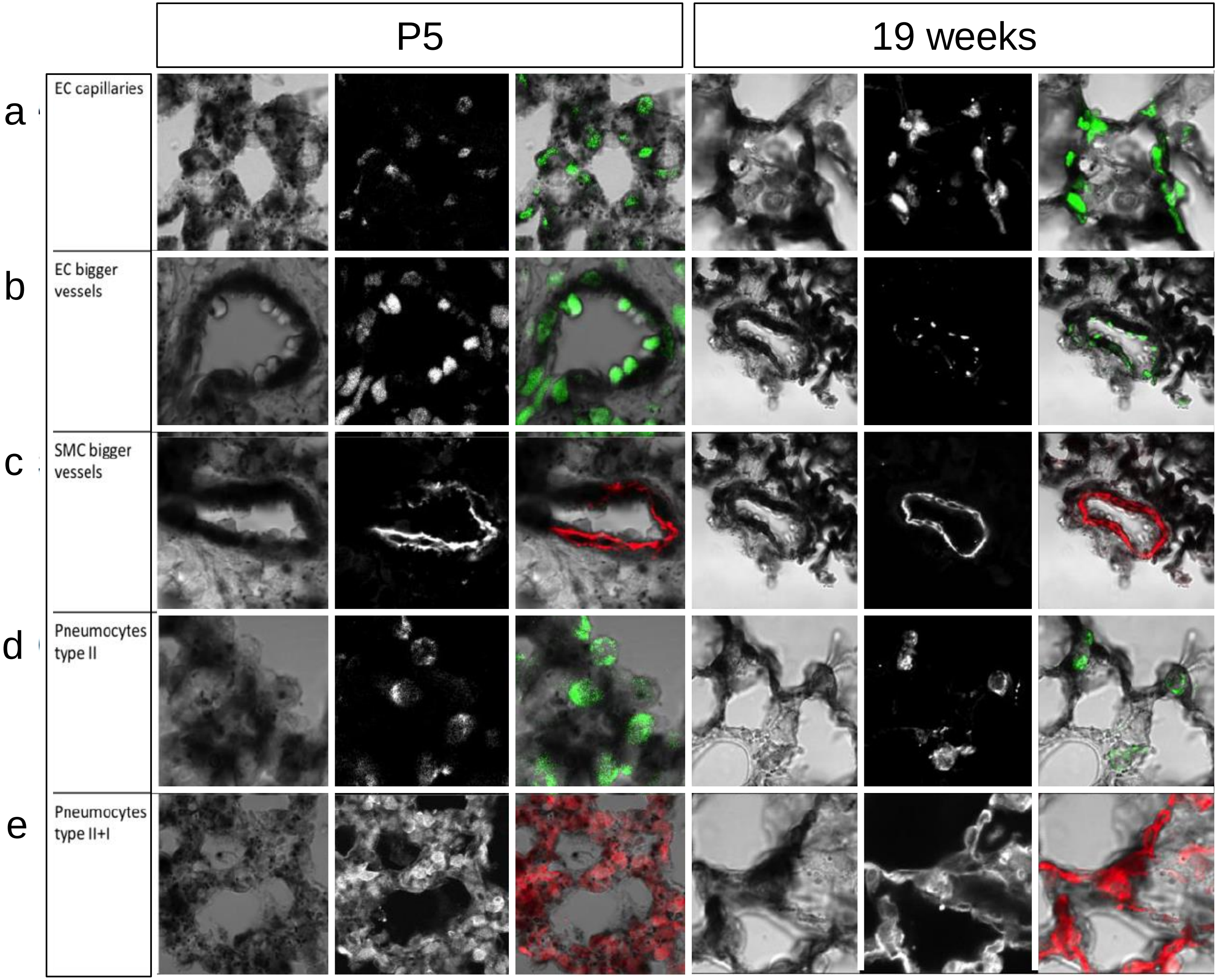

Egana et al. S4

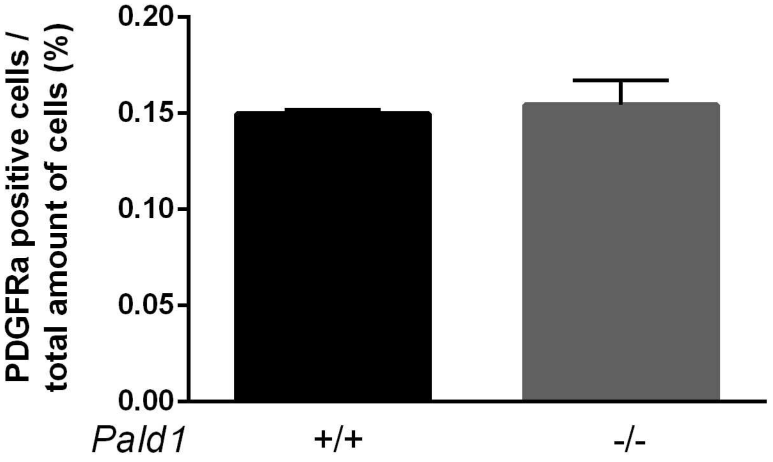

Egana et al. S5

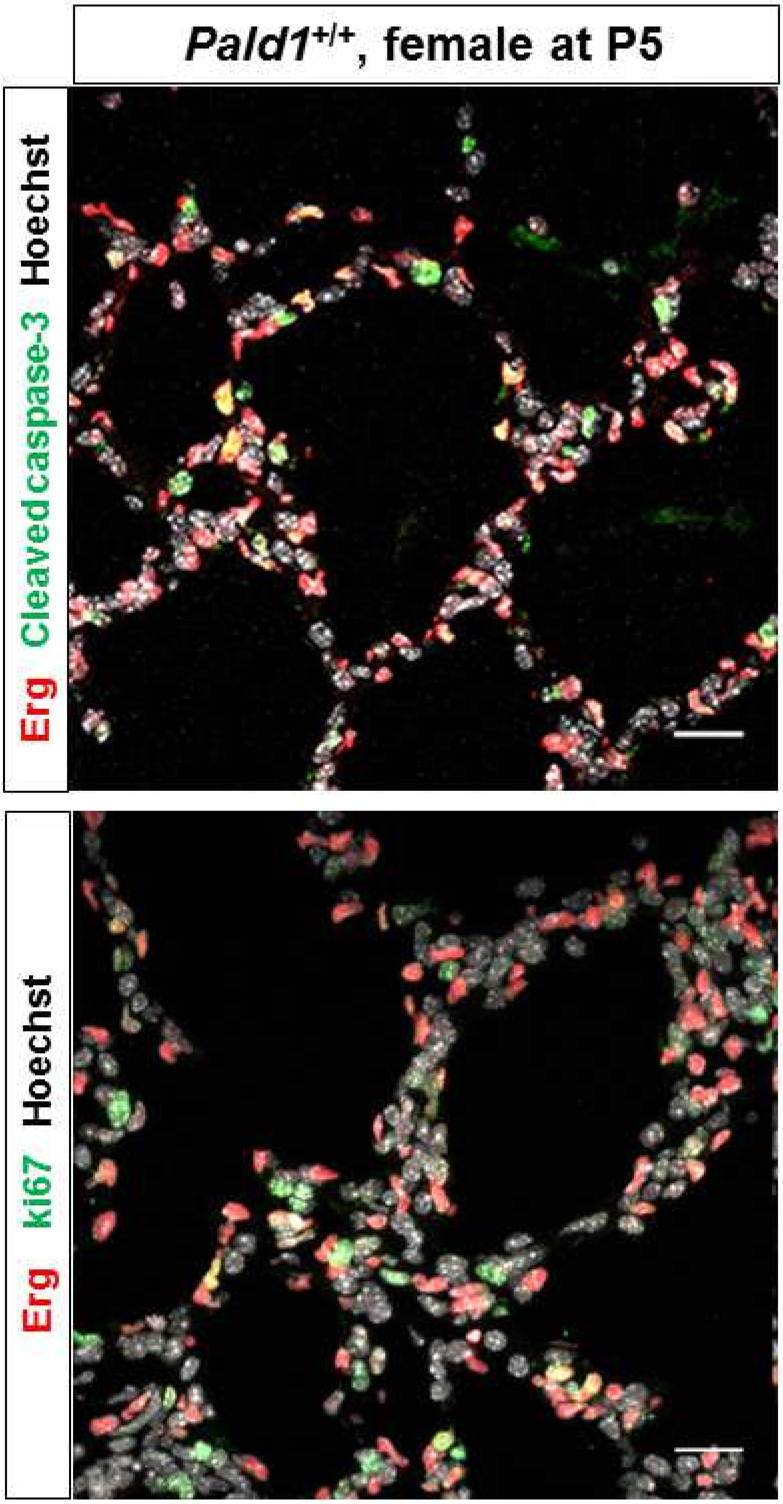

Egana et al. Figure S6

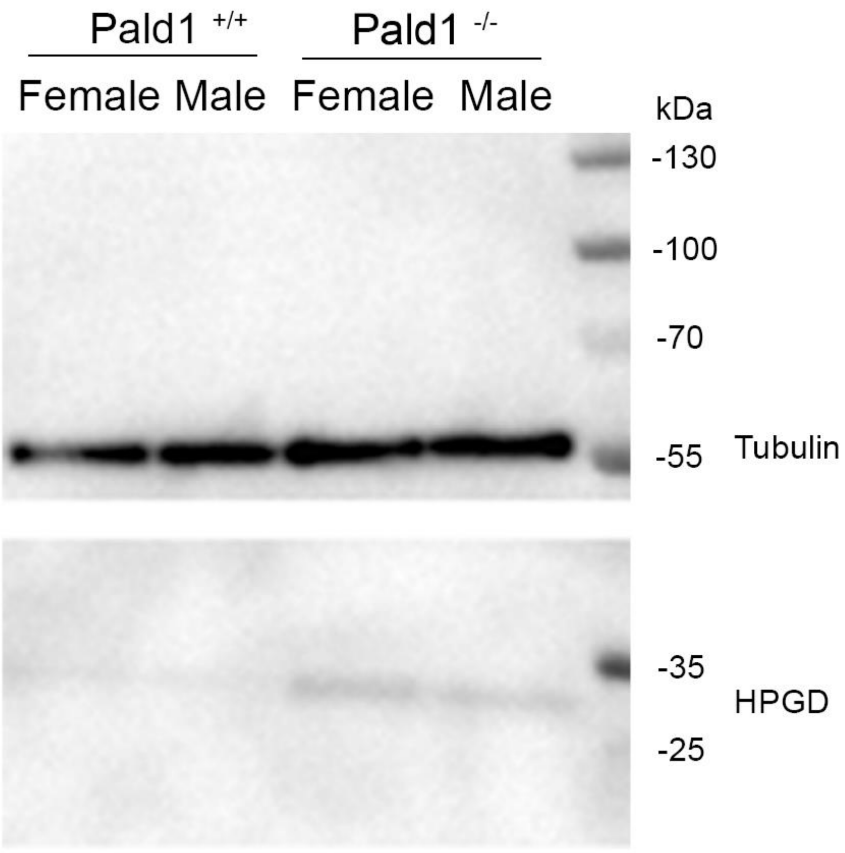

Egana et al. Figure S7

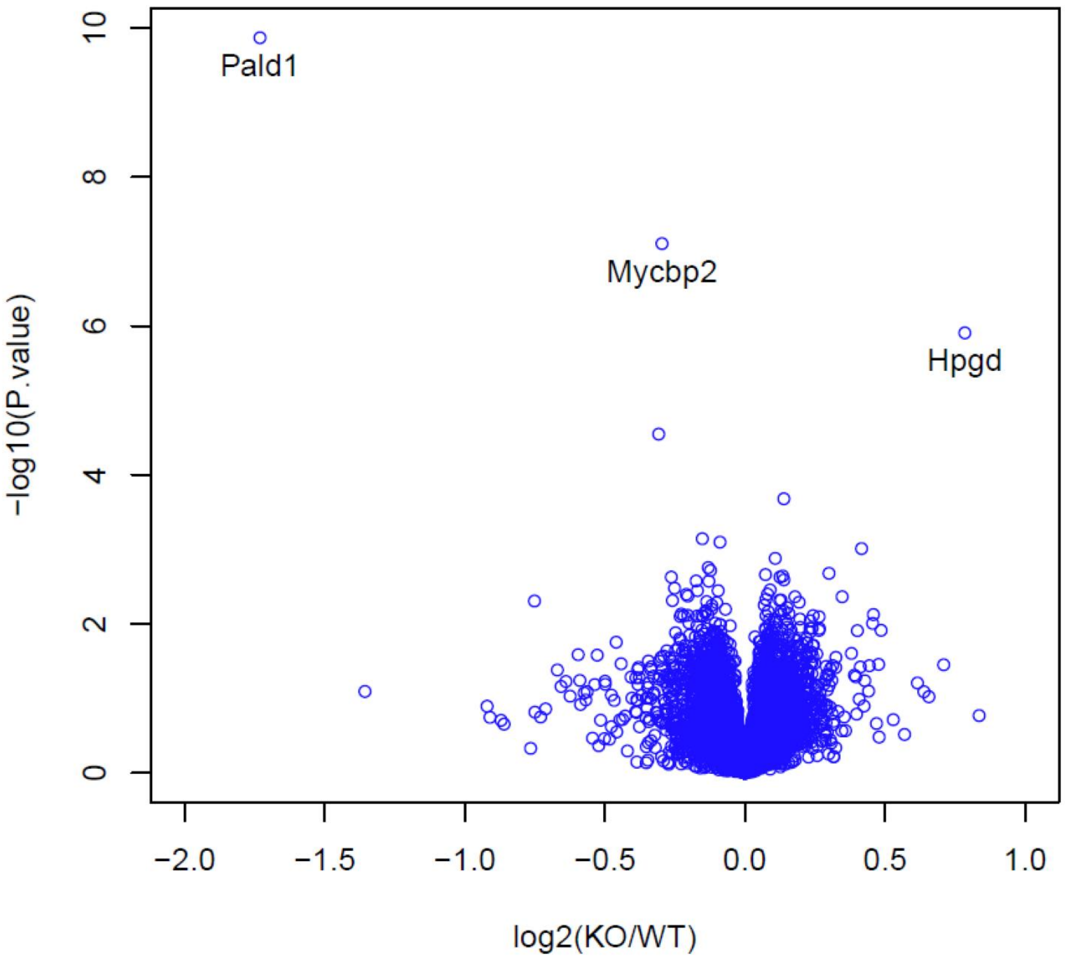

Supplement: Supplementary file 1 — Supplementary Information [file 41598_2017_14894_MOESM1_ESM.pdf]
